# Supplementary material for: A Subunit of the COP9 Signalosome, MoCsn6, Is Involved in Fungal Development, Pathogenicity, and Autophagy in Rice Blast Fungus
Source: Microbiol Spectr. 2022 Nov 29;10(6):e02020-22. doi: 10.1128/spectrum.02020-22 (PMC9769505; doi:10.1128/spectrum.02020-22)
Supplement: Supplemental file 1 — Supplemental material. Download spectrum.02020-22-s0001.pdf, PDF file, 1.3 MB [file spectrum.02020-22-s0001.pdf]

Table S1. List of genes related to autophagy screened in the yeast library of the whole genome of *M. oryzae*.

Gene ID	Gene description in NCBI	Strain	Virulence
MGG_13188	Voltage-gated potassium channel subunit beta-2	70-15	Normal
MGG_03853	Aldose 1-epimerase	70-15	Normal
MGG_01732	3-carboxymuconate cyclase	70-15	Normal
MGG_06315	transcription regulatory protein SNF5	70-15	Normal
MGG_08098	AP-3 adaptor complex subunit mu	70-15	Normal
MGG_04617	hypothetical protein	70-15	Normal
MGG_11750	hypothetical protein	70-15	Normal
MGG_04190	hypothetical protein	70-15	Normal
MGG_10844	hypothetical protein	70-15	Normal
MGG_17371	hypothetical protein	70-15	Normal
MGG_01432	hypothetical protein	70-15	Reduction
MGG_07038	hypothetical protein	70-15	Normal
MGG_01351	protein phosphatase 2C Ptc2	70-15	Normal
MGG_14219	ubiquitin thioesterase	70-15	Normal
MGG_03646	serine/threonine-protein phosphatase dullard	70-15	Reduction
MGG_06035	FK506-binding protein 1B	70-15	Normal
MGG_06747	glutathione S-transferase	70-15	Normal
MGG_00518	proliferating cell nuclear antigen	70-15	Normal
MGG_00476	xaa-Pro aminopeptidase 1	70-15	Normal
MGG_01041	cellulose signaling associated protein ENVOY	70-15	Normal
MGG_01592	eukaryotic translation initiation factor 2 subunit gamma	70-15	Normal
MGG_03244	V-type proton ATPase subunit B	70-15	Normal
MGG_04107	SET domain-containing protein 8	70-15	Normal
MGG_06099	serine/threonine-protein phosphatase PP2A catalytic subunit	70-15	Normal
MGG_06167	phytase	70-15	Normal
MGG_08862	dolichol-phosphate mannosyltransferase	70-15	Normal
MGG_09530	dipeptidase 1	70-15	Normal
MGG_10189	beta-glucosidase A	70-15	Normal
MGG_10506	glyoxalase	70-15	Normal
MGG_11400	endo-1,4-beta-xylanase D	70-15	Normal
MGG_12615	rab protein geranylgeranyltransferase component A	70-15	Normal
MGG_00588	CIA30 family protein	70-15	Normal
MGG_01135	cytochrome c oxidase polypeptide IV	70-15	Normal
MGG_03773	hydroxyquinol 1,2-dioxygenase	70-15	Normal
MGG_05992	sulfite oxidase	70-15	Normal
MGG_06860	coatomer subunit beta	70-15	Normal
MGG_07927	endochitinase 1	70-15	Normal

MGG_15739	inorganic pyrophosphatase	70-15	Normal
MGG_02626	septin	70-15	Normal
MGG_02647	UVI-1	70-15	Normal
MGG_04623	cation efflux family protein	70-15	Normal
MGG_05693	MIF domain-containing protein	70-15	Normal
MGG_09493	AP-2 complex subunit alpha	70-15	Normal
MGG_00471	exocyst complex component Sec15	70-15	Normal
MGG_01084	glyceraldehyde-3-phosphate dehydrogenase	70-15	Normal
MGG_02459	DNA polymerase delta small subunit	70-15	Normal
MGG_16154	tRNA-pseudouridine synthase,	70-15	Normal
MGG_03337	endoprotease	70-15	Normal
MGG_03692	glutaminyl-peptide cyclotransferase	70-15	Normal
MGG_03743	IQ calmodulin-binding domain-containing protein	70-15	Normal
MGG_04334	FAD binding domain-containing protein	70-15	Normal
MGG_04337	catalase-peroxidase 1	70-15	Normal
MGG_09664	beta-mannosidase	70-15	Normal
MGG_11543	lactoylglutathione lyase	70-15	Normal
MGG_05663	carboxypeptidase Y	70-15	Normal
MGG_06475	alpha-actinin	70-15	Normal
MGG_06519	WD domain-containing protein	70-15	Normal
MGG_07051	neutral ceramidase	70-15	Normal
MGG_08768	methyltransferase-UbiE family protein	70-15	Normal
MGG_09080	arginyl-tRNA synthetase	70-15	Normal
MGG_10569	pyruvate dehydrogenase E1 component subunit beta	70-15	Normal
MGG_12137	lysine decarboxylase	70-15	Normal
MGG_13246	amine oxidase	70-15	Normal
MGG_14878	thymocyte nuclear protein 1	70-15	Normal
MGG_00966	hypothetical protein	70-15	Normal
MGG_03601	alpha-galactosidase	70-15	Normal
MGG_05956	glutamyl-tRNA synthetase	70-15	Normal
MGG_08985	beta-xylosidase	70-15	Normal
MGG_06417	peptidyl-prolyl cis-trans isomerase cyp8	70-15	Normal
MGG_07148	ribose-phosphate pyrophosphokinase 3	70-15	Normal
MGG_12173	hydrolase	70-15	Normal
MGG_12726	phenazine biosynthesis protein PhzF family protein	70-15	Normal
MGG_13535	nucleoporin POM152	70-15	Normal
MGG_00998	lipase/esterase	70-15	Normal
MGG_01604	epidermal retinal dehydrogenase 2	70-15	Normal
MGG_04227	WD repeat-containing protein	70-15	Normal

Table S2 Primers used in this study

Primer name	Primer
**Primers for gene knock out and complementation**	
CSN6-Up-F	AGGCTAACTGACACTCTAGACATAGTTCCTTGGGCTCGGT
CSN6-Up-R	TGTTGACCTCCACTATCTATTCGCAGGCTCTCGGT
CSN6-Down-F	GGAATAGAGTAGATGCTATCTGCCTTTTTGGGT
CSN6-Down-R	CGACGGCCAGTGCCAAGCTTTCGACCGACTACTTTGGGC
CSN6-Long-F	TTTCCTCACTGGAGGAACATG
Long-HPH-R	GTCGGAGACGCTGTCGAACTT
CSN6-SF	CGACTCTGAGCTCCATGT
CSN6-SR	GTAGAGCGGCAGACTCGTTGAC
CSN6C-GFP-H3-F	ATCACAATGGCCGGATCC ATGGCTTCAGAAATGGAGACCA
CSN6C-GFP-H3-R	CTTGCTCACCATCCCGGG CATGATATCGCCCGCGGTAGAC
HPH-F	TAGTGGAGGTCAACAATGAATG
HPH-R	CATCTACTCTATTCCTTTGCCC
Tubulin-F	CCATCCCGAGCTTGTTGATA
Tubulin-R	GTAGTTCAGGTCACCGTATGAG
**Primer for yeast two hybrid**	
CSN1-AD-F	ATGGAGGCCAGTGAATTCATGGCGACTCCTCATGAGAAGC
CSN1-AD-R	CTCGAGCTCGATGGATCCAAATACGGATGCCATCGCTGAA
CSN2-AD-F	ATGGAGGCCAGTGAATTCATGTCCGACGACGACTTCATGC
CSN2-AD-R	CTCGAGCTCGATGGATCCTGTAAAGGTCGAATGAACTGCC
CSN3-AD-F	ATGGAGGCCAGTGAATTCATGGATCACTGCGCGTCGGTCC
CSN3-AD-R	CTCGAGCTCGATGGATCCCTGCCCAGATACTATGCCGGAC
CSN4-AD-F	ATGGAGGCCAGTGAATTCATGGCCTCCGACTCGATAAAGG
CSN4-AD-R	CTCGAGCTCGATGGATCCGACTACCAAGTTGGCCGCAACA
CSN5-AD-F	ATGGAGGCCAGTGAATTCATGGATGTTGCTATGAAGTCGT
CSN5-AD-R	CTCGAGCTCGATGGATCCCGACGCAGCCGACGGCTCAGCC
CSN6-AD-F	GGAGGCCAGTGAATTCATGGCTTCAGAAATGGAG
CSN6-AD-R	CGAGCTCGATGGATCCCGACATGATATCGCCCGC
CSN7a-AD-F	ATGGAGGCCAGTGAATTCATGGAGCAAGCAAAGGCTCTCA
CSN7a-AD-R	CTCGAGCTCGATGGATCCAAGCTTTCTTCGACTCGACCTC
CSN1-BD-F	GCCATGGAGGCCGAATTCATGGCGACTCCTCATGAGAAGC
CSN1-BD-R	CTGCAGGTCGACGGATCCAAATACGGATGCCATCGCTGAA
CSN2-BD-F	GCCATGGAGGCCGAATTCATGTCCGACGACGACTTCATGC
CSN2-BD-R	CTGCAGGTCGACGGATCCTGTAAAGGTCGAATGAACTGCC
CSN3-BD-F	GCCATGGAGGCCGAATTCATGGATCACTGCGCGTCGGTCC
CSN3-BD-R	CTGCAGGTCGACGGATCCCTGCCCAGATACTATGCCGGAC
CSN4-BD-F	GCCATGGAGGCCGAATTCATGGCCTCCGACTCGATAAAGG
CSN4-BD-R	CTGCAGGTCGACGGATCCGACTACCAAGTTGGCCGCAACA
CSN5-BD-F	GCCATGGAGGCCGAATTCATGGATGTTGCTATGAAGTCGT
CSN5-BD-R	CTGCAGGTCGACGGATCCCGACGCAGCCGACGGCTCAGCC
CSN6-BD-F	CATGGAGGCCGAATTCATGGCTTCAGAAATGGAGACC
CSN6-BD-R	CTGCAGGTCGACGGATCCCGACATGATATCGCCCGC
CSN7a-BD-F	GCCATGGAGGCCGAATTCATGGAGCAAGCAAAGGCTCTCA
CSN7a-BD-R	CTGCAGGTCGACGGATCCAAGCTTTCTTCGACTCGACCTC
ATG6-BD-F	GCCATGGAGGCCGAATTCATGATGTTTTGCCAAAAATGCC

ATG6-BD-R	CTGCAGGTCGACGGATCCGGTCGAGCTTGAGCCCAAAACC
**Primer for Co-IP**	
ATG6-GFP-F	ATCACAATGGCCGGATCC ATGATGTTTTGCCAAAAATGCC
ATG6-GFP-R	CTTGCTCACCATCCCGGG GGTCGAGCTTGAGCCCAAAACC
CSN6-FLAG-F	ATAGAGTAGATGGAATTCGATGACATTCATGGCGATGTAT
CSN6-FLAG-R	GTCCCCGGGGATGGATCCCGACATGATATCGCCCGCGGTA
CSN1-GFP-F	ATCACAATGGCCGGATCCATGGCGACTCCTCATGAGAAGC
CSN1-GFP-R	CTTGCTCACCATCCCGGGAAATACGGATGCCATCGCTGAA
CSN3-GFP-F	ATCACAATGGCCGGATCCATGGATCACTGCGCGTCGGTCC
CSN3-GFP-R	CTTGCTCACCATCCCGGGCTGCCCAGATACTATGCCGGAC
CSN4-GFP-F	ATCACAATGGCCGGATCCATGGCCTCCGACTCGATAAAGG
CSN4-GFP-R	CTTGCTCACCATCCCGGGGACTACCAAGTTGGCCGCAACA
CSN5-GFP-F	ATCACAATGGCCGGATCCATGGATGTTGCTATGAAGTCGT
CSN5-GFP-R	CTTGCTCACCATCCCGGGCGACGCAGCCGACGGCTCAGCC
CSN7a-GFP-F	ATCACAATGGCCGGATCCATGGAGCAAGCAAAGGCTCTCA
CSN7a-GFP-R	CTTGCTCACCATCCCGGGAAGCTTTCTTCGACTCGACCTC
**Primer for colocalization**	
H_2B-mCherry-F	ATGGTCGGATCCATCCCCGGGATGCCCCCCAAGGCCGCTGACAA
H_2B-mCherry-R	TTACTGCAGGTCGACTCTAGAGCCGCCGGTGGAGTGGCGGCC
CSN6-GFP-F	ACAATCACTAGTGAATTC GATGACATTCATGGCGATGTAT
CSN6-GFP-R	CATCCCGGGGATGGATCC CATGATATCGCCCGCGGTAGAC
**Primer for quantitative real-time PCR**	
RT-HPH-F	ATGTCCTGCGGGTAAATAGC
RT-HPH-R	GATGCAATAGGTCAGGCTCTC
RT-Tubulin-F	ACAACTTCGTCTTCGGTCAG
RT-Tubulin-R	GTGATCTGGAAACCCTGGAG

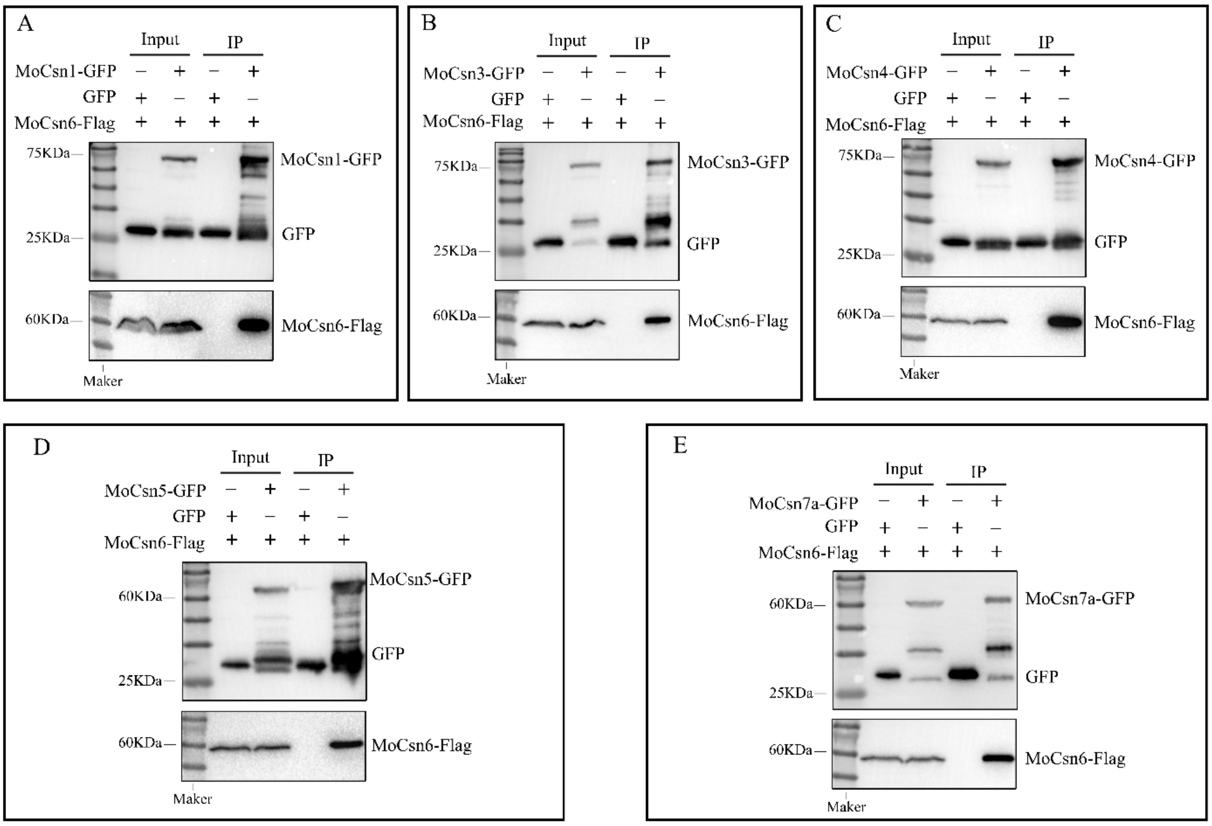

Figure S1 The interactions between MoCsn6 and MoCsn1, MoCsn3, MoCsn4, MoCsn5, and MoCsn7a were detected by Co-IP. MoCsn1-GFP, MoCsn3-GFP, MoCsn4-GFP, MoCsn5-GFP and MoCsn7a-GFP were constructed and transformed into the 70-15 strain with MoCsn6-3×Flag. Flag antibody and GFP antibody were used to detect the expression of the GFP label in transformants by western blotting. A. MoCsn6 interacts with MoCsn1 *in vivo*. B. MoCsn6 interacts with MoCsn3 *in vivo*. C. MoCsn6 interacts with MoCsn4 *in vivo*. D. MoCsn6 interacts with MoCsn5 *in vivo*. E. MoCsn6 interacts with MoCsn7a *in vivo*.

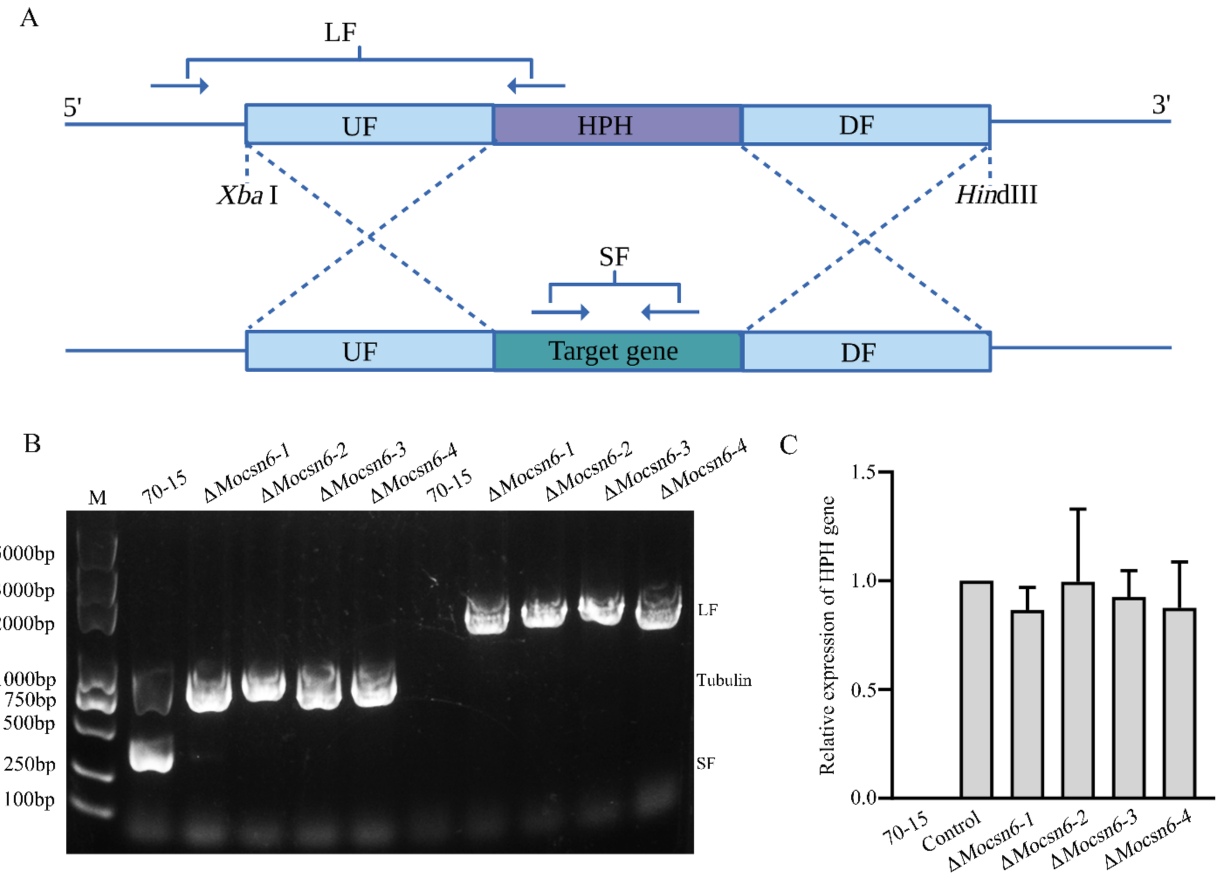

Figure S2 Identification of knockout mutants. A. The knockout model in *M. oryzae*. B. Knockout event of Δ*Mocsn6* mutants verified by PCR. A 1.2–2.0-kb fragment marked as a knockout event was amplified in the mutants but not in the 70-15 strain. A 500-bp characteristic band of the target gene was amplified in the 70-15 strain but not in the mutants. Tubulin was used as a positive control. C. The insertion copy number of the deletion mutants was verified by quantitative real-time PCR. Tubulin was used as an internal reference. The primers used in this experiment are shown in Table S2.

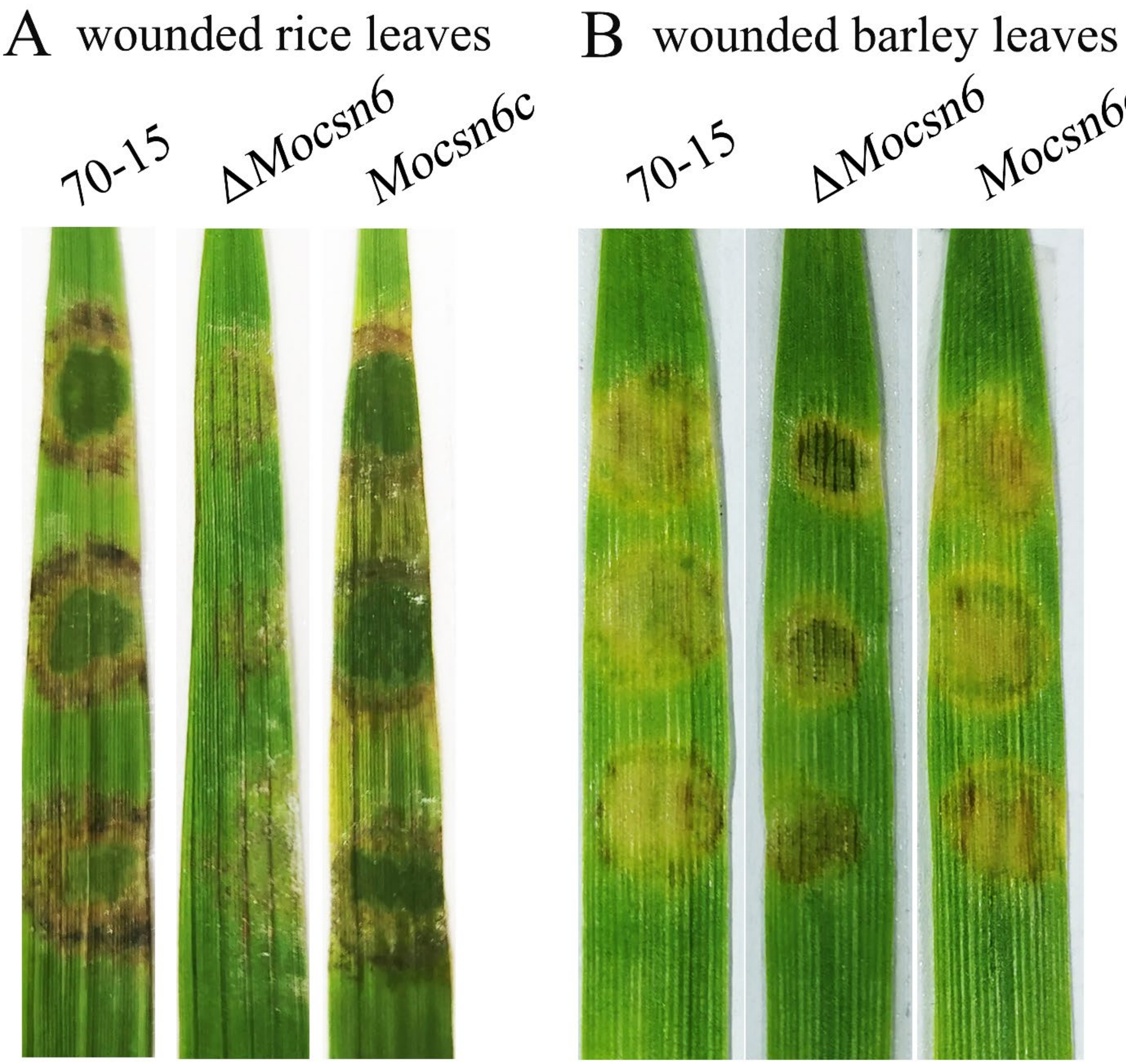

Figure S3 MoCsn6 is also required for pathogenicity when inoculated on wounded barley and rice leaves. A. Disease symptoms on wounded leaves of rice inoculated with mycelial plugs of 70-15, Δ*Mocsn6* mutant and *Mocsn6c.* B. Disease symptoms on wounded leaves of barley inoculated with mycelial plugs of 70-15, Δ*Mocsn6* mutant and *Mocsn6c.*

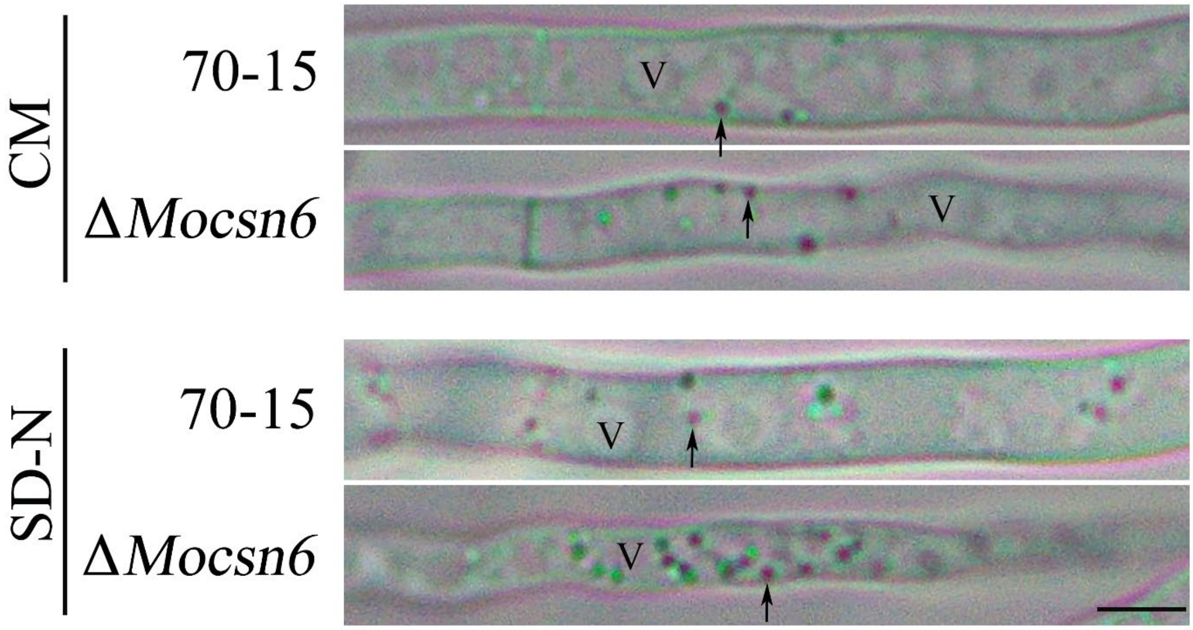

Figure S4 Autophagy was enhanced in the Δ*Mocsn6* mutant. The cytoplasm was filled with autophagosomes in the Δ*Mocsn6* mutant, whereas a few autophagosomes were in the cytoplasm of 70-15 after culturing on CM medium for 2 days. After 3 hours of SD-N induction, autophagosomes were increased in both 70-15 and Δ*Mocsn6* but were increased more in Δ*Mocsn6* than in 70-15. V, vacuole. An autophagosome is indicated by the arrow. Scale bar, 5 μm.
